# *N*-(5-Ethylsulfanyl-1,3,4-thiadiazol-2-yl)-2-(4,5,6,7-tetrahydrothieno[3,2-*c*]pyri­din-5-yl)acetamide

**DOI:** 10.1107/S1600536811017107

**Published:** 2011-05-25

**Authors:** Shuang Zhi, Shuai Mu, Ying Liu, Deng-Ke Liu

**Affiliations:** a School of Environmental and Chemical Engineering, Tianjin Polytechnic University, Tianjin 300160, People’s Republic of China; b School of Chemical Engineering and Technology, Tianjin University, Tianjin, 300072, People’s Republic of China; c Tianjin Institute of Pharmaceutical Research, Tianjin, 300193, People’s Republic of China

## Abstract

In the title compound, C_13_H_16_N_4_OS_3_, a thienopyridine­derivative, the tetra­hydro­pyridine ring exhibits a half-chair conformation, and the folded conformation of the mol­ecule is defined by the N—C—C—N torsion angle of −78.85 (16)°. The crystal packing features inter­molecular C—H⋯N, N—H⋯N and C—H⋯O hydrogen bonds.

## Related literature

The title compound is a potential anti­platelet agent. As irreversible P2Y12 antagonists, thienopyridines have proved the relevance of inhibiting signaling *via* the platelet-specific P2Y12 ADP receptor in the prevention of cardiovascular events, see: Iyengar (2009 ▶); Franchini & Mannucci, (2009 ▶); Van Giezen *et al.* (2009 ▶); Van Giezen & Humphries (2005 ▶). For a related structure, see: Chen *et al.* (2010 ▶). For the synthesis of the title compound, see: Liu *et al.* (2008 ▶).
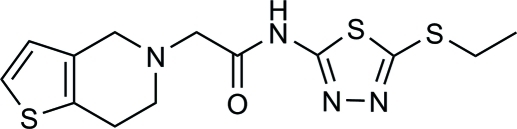


## Experimental

### 

#### Crystal data

C_13_H_16_N_4_OS_3_
*M* *_r_* = 340.48
Monoclinic, 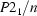

*a* = 6.532 (4) Å
*b* = 9.788 (6) Å
*c* = 23.491 (15) Å
β = 95.524 (6)°
*V* = 1494.8 (16) Å^3^
*Z* = 4
Mo *K*α radiation
μ = 0.50 mm^−1^
*T* = 113 K
0.28 × 0.22 × 0.18 mm

#### Data collection

Rigaku Saturn CCD area-detector diffractometer
Absorption correction: multi-scan (*CrystalClear*; Rigaku/MSC, 2005 ▶) *T*_min_ = 0.873, *T*_max_ = 0.916
12423 measured reflections
3545 independent reflections
2653 reflections with *I* > 2σ(*I*)
*R*_int_ = 0.036

#### Refinement

*R*[*F* ^2^ > 2σ(*F* ^2^)] = 0.030
*wR*(*F* ^2^) = 0.079
*S* = 1.03
3545 reflections
195 parameters
1 restraint
H atoms treated by a mixture of independent and constrained refinement
Δρ_max_ = 0.44 e Å^−3^
Δρ_min_ = −0.23 e Å^−3^

### 

Data collection: *CrystalClear* (Rigaku/MSC, 2005 ▶); cell refinement: *CrystalClear*; data reduction: *CrystalClear*; program(s) used to solve structure: *SHELXS97* (Sheldrick, 2008 ▶); program(s) used to refine structure: *SHELXL97* (Sheldrick, 2008 ▶); molecular graphics: *SHELXTL* (Sheldrick, 2008 ▶); software used to prepare material for publication: *CrystalStructure* (Rigaku/MSC, 2005 ▶).

## Supplementary Material

Crystal structure: contains datablocks global, I. DOI: 10.1107/S1600536811017107/kp2327sup1.cif

Structure factors: contains datablocks I. DOI: 10.1107/S1600536811017107/kp2327Isup2.hkl

Supplementary material file. DOI: 10.1107/S1600536811017107/kp2327Isup3.cdx

Additional supplementary materials:  crystallographic information; 3D view; checkCIF report

## Figures and Tables

**Table 1:** Hydrogen-bond geometry (Å, °)

*D*—H⋯*A*	*D*—H	H⋯*A*	*D*⋯*A*	*D*—H⋯*A*
C12—H12*B*⋯N1^i^	0.99	2.60	3.473 (3)	147
N2—H2⋯N3^ii^	0.89 (1)	2.02 (1)	2.902 (2)	171 (2)
C5—H5⋯O1^iii^	0.95	2.46	3.279 (2)	145

Symmetry codes: (i) 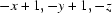; (ii) 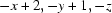; (iii) 
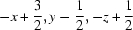
.

**Fig. 1.**
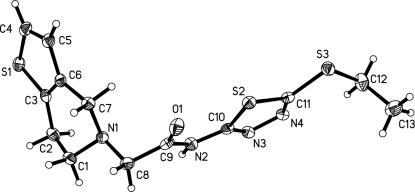
The molecular structure of (I), with the atom-numbering scheme and 50% probability displacement ellipsoids.

**Fig. 2.**
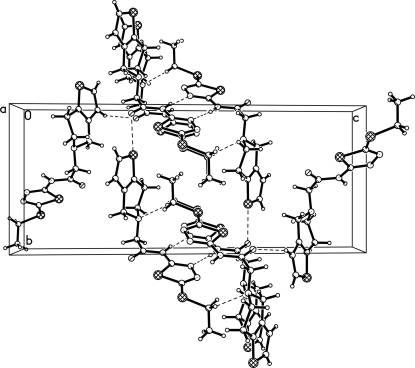
Packing diagram for (I) with hydrogen bonds drawn as dashed lines.

## supplementary crystallographic information

### Comment

As irreversible P2Y12 antagonists, the thienopyridines (*e.g.*, clopidogrel and prasugrel) have been further proved the relevance of inhibiting signaling *via* the platelet-specific P2Y12 ADP receptor in the prevention of cardiovascular events (Iyengar, 2009; Van Giezen & Humphries, 2005; Franchini, *et al.*, 2009). The structure of the title compound (I), a new derivative of thienopyridine, is presented here.

The tetrahydropyridine ring is in a half-chair conformation (Fig. 1). The thiadiazole ring plane (r.m.s. deviation 0.0020Å) and the acidamide plane (r.m.s. deviation 0.0074 Å) are almost coplanar, with a dihedral angle of 3.24 (9)°. The dihedral angles formed between the thiadiazole ring plane and the thiophene ring plane, the acidamide plane and the thiophene ring plane are 76.19 (6)°  and 78.47 (7)° , respectively. Crystal packing is via hydrogen bonds C—H···N, N—H···N and C—H···O (Table 1, Fig. 2).

### Experimental

Chloracetyl chloride was dropwised added into a mixture of 5-(ethylthio)-1,3,4-thiadiazol-2-amine, TEA and DMF at 268 K. After stirred for 3 h, the mixture was poured into cold water. 2-Chloro-*N*-(5-(ethylthio)-1,3,4-thiadiazol-2-yl)acetamide was precipitated as an intermediate. Then the intermediate, equimolar quantities thienopyridine salt and TEA were refluxed for 5 h in acetonitrile, and the product was obtained by silica gel column chromatography. Crystallisation of the obtained yellow solid from methanol afforded light-yellow crystals suitable for X-ray analysis.

### Refinement

The N–H bond was restrained to 0.90 Å, and other H atoms were positioned geometrioncally and refined using a riding model, with d(C—H)=0.95–0.99 Å, and *U*_iso_(H)=1.2*U*_eq_(C) of the parent atom.

### Crystal data

**Table:**

C_13_H_16_N_4_OS_3_	*F*(000) = 712
*M**_r_* = 340.48	*D*_x_ = 1.513 Mg m^−^^3^
Monoclinic, *P*2_1_/*n*	Mo *K*α radiation, λ = 0.71073 Å
Hall symbol: -P 2yn	Cell parameters from 4582 reflections
*a* = 6.532 (4) Å	θ = 1.7–27.9°
*b* = 9.788 (6) Å	µ = 0.50 mm^−^^1^
*c* = 23.491 (15) Å	*T* = 113 K
β = 95.524 (6)°	Prism, colourless
*V* = 1494.8 (16) Å^3^	0.28 × 0.22 × 0.18 mm
*Z* = 4	

### Data collection

**Table:**

Rigaku Saturn CCD area-detector diffractometer	3545 independent reflections
Radiation source: rotating anode	2653 reflections with *I* > 2σ(*I*)
multilayer	*R*_int_ = 0.036
Detector resolution: 14.63 pixels mm^-1^	θ_max_ = 27.9°, θ_min_ = 1.7°
ω and φ scans	*h* = −8→8
Absorption correction: multi-scan (*CrystalClear*; Rigaku/MSC, 2005)	*k* = −12→12
*T*_min_ = 0.873, *T*_max_ = 0.916	*l* = −28→30
12423 measured reflections	

### Refinement

**Table:**

Refinement on *F*^2^	Primary atom site location: structure-invariant direct methods
Least-squares matrix: full	Secondary atom site location: difference Fourier map
*R*[*F*^2^ > 2σ(*F*^2^)] = 0.030	Hydrogen site location: inferred from neighbouring sites
*wR*(*F*^2^) = 0.079	H atoms treated by a mixture of independent and constrained refinement
*S* = 1.03	*w* = 1/[σ^2^(*F*_o_^2^) + (0.042*P*)^2^] where *P* = (*F*_o_^2^ + 2*F*_c_^2^)/3
3545 reflections	(Δ/σ)_max_ = 0.004
195 parameters	Δρ_max_ = 0.44 e Å^−^^3^
1 restraint	Δρ_min_ = −0.23 e Å^−^^3^

### Special details

**Table:**

Geometry. All e.s.d.'s (except the e.s.d. in the dihedral angle between two l.s. planes) are estimated using the full covariance matrix. The cell e.s.d.'s are taken into account individually in the estimation of e.s.d.'s in distances, angles and torsion angles; correlations between e.s.d.'s in cell parameters are only used when they are defined by crystal symmetry. An approximate (isotropic) treatment of cell e.s.d.'s is used for estimating e.s.d.'s involving l.s. planes.
Refinement. Refinement of *F*^2^ against ALL reflections. The weighted *R*-factor *wR* and goodness of fit *S* are based on *F*^2^, conventional *R*-factors *R* are based on *F*, with *F* set to zero for negative *F*^2^. The threshold expression of *F*^2^ > σ(*F*^2^) is used only for calculating *R*-factors(gt) *etc*. and is not relevant to the choice of reflections for refinement. *R*-factors based on *F*^2^ are statistically about twice as large as those based on *F*, and *R*- factors based on ALL data will be even larger.

### Fractional atomic coordinates and isotropic or equivalent isotropic displacement parameters (Å^2^)

**Table:**

	*x*	*y*	*z*	*U*_iso_*/*U*_eq_	
S1	1.09432 (6)	−0.17109 (4)	0.167990 (17)	0.02366 (11)	
S2	0.59937 (6)	0.64557 (4)	0.091056 (16)	0.01921 (10)	
S3	0.24260 (6)	0.77190 (4)	0.021966 (17)	0.02324 (11)	
O1	0.87853 (17)	0.54420 (11)	0.17006 (4)	0.0257 (3)	
N1	1.16286 (18)	0.27779 (12)	0.15327 (5)	0.0188 (3)	
N2	0.9587 (2)	0.51028 (13)	0.08012 (5)	0.0195 (3)	
N3	0.76496 (19)	0.59529 (13)	−0.00107 (5)	0.0199 (3)	
N4	0.58266 (19)	0.66332 (13)	−0.01858 (5)	0.0196 (3)	
C1	1.3598 (2)	0.20466 (16)	0.15877 (7)	0.0220 (3)	
H1A	1.4181	0.2046	0.1993	0.026*	
H1B	1.4584	0.2513	0.1359	0.026*	
C2	1.3270 (2)	0.05905 (16)	0.13799 (7)	0.0233 (3)	
H2A	1.2955	0.0577	0.0959	0.028*	
H2B	1.4531	0.0045	0.1479	0.028*	
C3	1.1520 (2)	0.00006 (15)	0.16615 (6)	0.0194 (3)	
C4	0.8868 (2)	−0.14081 (15)	0.20530 (7)	0.0234 (3)	
H4	0.7989	−0.2103	0.2173	0.028*	
C5	0.8627 (2)	−0.00627 (15)	0.21566 (6)	0.0214 (3)	
H5	0.7562	0.0299	0.2360	0.026*	
C6	1.0153 (2)	0.07498 (15)	0.19257 (6)	0.0184 (3)	
C7	1.0281 (2)	0.22806 (15)	0.19494 (6)	0.0197 (3)	
H7A	0.8889	0.2676	0.1865	0.024*	
H7B	1.0824	0.2573	0.2338	0.024*	
C8	1.1928 (2)	0.42478 (15)	0.15876 (7)	0.0220 (3)	
H8A	1.3052	0.4538	0.1361	0.026*	
H8B	1.2324	0.4484	0.1993	0.026*	
C9	0.9969 (2)	0.49862 (14)	0.13783 (7)	0.0204 (3)	
C10	0.7907 (2)	0.57771 (14)	0.05403 (6)	0.0181 (3)	
C11	0.4826 (2)	0.69389 (15)	0.02453 (6)	0.0190 (3)	
C12	0.2098 (3)	0.84165 (17)	−0.04979 (7)	0.0300 (4)	
H12A	0.3070	0.7958	−0.0734	0.036*	
H12B	0.0686	0.8216	−0.0669	0.036*	
C13	0.2449 (3)	0.99201 (18)	−0.05141 (8)	0.0362 (4)	
H13A	0.1524	1.0379	−0.0271	0.054*	
H13B	0.2173	1.0249	−0.0908	0.054*	
H13C	0.3879	1.0121	−0.0374	0.054*	
H2	1.047 (2)	0.4714 (17)	0.0587 (7)	0.038 (5)*	

### Atomic displacement parameters (Å^2^)

**Table:**

	*U*^11^	*U*^22^	*U*^33^	*U*^12^	*U*^13^	*U*^23^
S1	0.0286 (2)	0.0204 (2)	0.0220 (2)	0.00424 (17)	0.00251 (17)	0.00018 (16)
S2	0.0206 (2)	0.02023 (18)	0.0177 (2)	0.00016 (15)	0.00653 (16)	−0.00083 (15)
S3	0.0217 (2)	0.0242 (2)	0.0244 (2)	0.00316 (16)	0.00550 (17)	0.00097 (16)
O1	0.0338 (7)	0.0255 (6)	0.0190 (6)	0.0047 (5)	0.0095 (5)	0.0034 (5)
N1	0.0177 (7)	0.0200 (6)	0.0189 (7)	0.0010 (5)	0.0038 (5)	0.0011 (5)
N2	0.0192 (7)	0.0222 (6)	0.0178 (7)	0.0017 (5)	0.0052 (5)	−0.0018 (5)
N3	0.0185 (7)	0.0228 (6)	0.0186 (7)	−0.0007 (5)	0.0030 (5)	−0.0029 (5)
N4	0.0179 (7)	0.0210 (6)	0.0200 (7)	−0.0015 (5)	0.0026 (5)	−0.0019 (5)
C1	0.0165 (8)	0.0283 (8)	0.0213 (8)	0.0002 (6)	0.0017 (6)	−0.0002 (6)
C2	0.0190 (8)	0.0286 (8)	0.0224 (8)	0.0039 (7)	0.0031 (7)	−0.0024 (7)
C3	0.0197 (8)	0.0213 (7)	0.0164 (8)	0.0025 (6)	−0.0016 (6)	0.0005 (6)
C4	0.0278 (9)	0.0243 (8)	0.0181 (8)	−0.0009 (7)	0.0020 (7)	0.0045 (6)
C5	0.0235 (8)	0.0252 (8)	0.0157 (8)	0.0019 (6)	0.0031 (6)	0.0020 (6)
C6	0.0198 (8)	0.0210 (7)	0.0141 (7)	0.0014 (6)	−0.0003 (6)	0.0012 (6)
C7	0.0198 (8)	0.0223 (7)	0.0174 (8)	0.0003 (6)	0.0042 (6)	−0.0009 (6)
C8	0.0216 (8)	0.0220 (8)	0.0223 (8)	−0.0037 (6)	0.0020 (7)	0.0002 (6)
C9	0.0248 (8)	0.0154 (7)	0.0216 (8)	−0.0056 (6)	0.0049 (7)	0.0010 (6)
C10	0.0191 (8)	0.0166 (7)	0.0195 (8)	−0.0025 (6)	0.0067 (6)	−0.0013 (6)
C11	0.0193 (8)	0.0176 (7)	0.0200 (8)	−0.0034 (6)	0.0024 (6)	−0.0011 (6)
C12	0.0335 (10)	0.0342 (9)	0.0216 (9)	0.0088 (8)	−0.0004 (7)	−0.0013 (7)
C13	0.0387 (11)	0.0375 (10)	0.0303 (10)	−0.0098 (8)	−0.0067 (8)	0.0095 (8)

### Geometric parameters (Å, °)

**Table:**

S1—C4	1.7096 (18)	C2—H2A	0.9900
S1—C3	1.7186 (18)	C2—H2B	0.9900
S2—C10	1.7232 (16)	C3—C6	1.352 (2)
S2—C11	1.7372 (18)	C4—C5	1.351 (2)
S3—C11	1.7396 (18)	C4—H4	0.9500
S3—C12	1.812 (2)	C5—C6	1.423 (2)
O1—C9	1.2179 (18)	C5—H5	0.9500
N1—C8	1.456 (2)	C6—C7	1.501 (2)
N1—C7	1.4614 (18)	C7—H7A	0.9900
N1—C1	1.467 (2)	C7—H7B	0.9900
N2—C9	1.359 (2)	C8—C9	1.510 (2)
N2—C10	1.373 (2)	C8—H8A	0.9900
N2—H2	0.885 (9)	C8—H8B	0.9900
N3—C10	1.300 (2)	C12—C13	1.490 (3)
N3—N4	1.3914 (18)	C12—H12A	0.9900
N4—C11	1.2921 (19)	C12—H12B	0.9900
C1—C2	1.515 (2)	C13—H13A	0.9800
C1—H1A	0.9900	C13—H13B	0.9800
C1—H1B	0.9900	C13—H13C	0.9800
C2—C3	1.491 (2)		
			
C4—S1—C3	91.75 (7)	C5—C6—C7	125.60 (13)
C10—S2—C11	85.86 (8)	N1—C7—C6	110.02 (11)
C11—S3—C12	102.89 (8)	N1—C7—H7A	109.7
C8—N1—C7	110.75 (11)	C6—C7—H7A	109.7
C8—N1—C1	111.43 (12)	N1—C7—H7B	109.7
C7—N1—C1	110.99 (12)	C6—C7—H7B	109.7
C9—N2—C10	123.21 (13)	H7A—C7—H7B	108.2
C9—N2—H2	117.7 (13)	N1—C8—C9	110.00 (13)
C10—N2—H2	119.1 (13)	N1—C8—H8A	109.7
C10—N3—N4	112.50 (12)	C9—C8—H8A	109.7
C11—N4—N3	111.23 (13)	N1—C8—H8B	109.7
N1—C1—C2	109.60 (13)	C9—C8—H8B	109.7
N1—C1—H1A	109.8	H8A—C8—H8B	108.2
C2—C1—H1A	109.8	O1—C9—N2	121.50 (15)
N1—C1—H1B	109.8	O1—C9—C8	122.81 (15)
C2—C1—H1B	109.8	N2—C9—C8	115.69 (13)
H1A—C1—H1B	108.2	N3—C10—N2	121.95 (13)
C3—C2—C1	108.17 (12)	N3—C10—S2	114.88 (12)
C3—C2—H2A	110.1	N2—C10—S2	123.17 (12)
C1—C2—H2A	110.1	N4—C11—S2	115.51 (12)
C3—C2—H2B	110.1	N4—C11—S3	126.62 (13)
C1—C2—H2B	110.1	S2—C11—S3	117.83 (9)
H2A—C2—H2B	108.4	C13—C12—S3	113.02 (12)
C6—C3—C2	124.21 (14)	C13—C12—H12A	109.0
C6—C3—S1	111.17 (12)	S3—C12—H12A	109.0
C2—C3—S1	124.61 (11)	C13—C12—H12B	109.0
C5—C4—S1	111.89 (12)	S3—C12—H12B	109.0
C5—C4—H4	124.1	H12A—C12—H12B	107.8
S1—C4—H4	124.1	C12—C13—H13A	109.5
C4—C5—C6	112.24 (14)	C12—C13—H13B	109.5
C4—C5—H5	123.9	H13A—C13—H13B	109.5
C6—C5—H5	123.9	C12—C13—H13C	109.5
C3—C6—C5	112.94 (14)	H13A—C13—H13C	109.5
C3—C6—C7	121.45 (13)	H13B—C13—H13C	109.5
			
C10—N3—N4—C11	0.50 (17)	C7—N1—C8—C9	−70.74 (16)
C8—N1—C1—C2	−165.54 (12)	C1—N1—C8—C9	165.18 (12)
C7—N1—C1—C2	70.53 (16)	C10—N2—C9—O1	2.6 (2)
N1—C1—C2—C3	−49.63 (17)	C10—N2—C9—C8	−178.09 (13)
C1—C2—C3—C6	16.2 (2)	N1—C8—C9—O1	100.49 (17)
C1—C2—C3—S1	−165.28 (12)	N1—C8—C9—N2	−78.85 (16)
C4—S1—C3—C6	−0.65 (13)	N4—N3—C10—N2	178.63 (12)
C4—S1—C3—C2	−179.37 (14)	N4—N3—C10—S2	−1.53 (16)
C3—S1—C4—C5	0.16 (13)	C9—N2—C10—N3	175.29 (14)
S1—C4—C5—C6	0.36 (18)	C9—N2—C10—S2	−4.5 (2)
C2—C3—C6—C5	179.69 (14)	C11—S2—C10—N3	1.57 (12)
S1—C3—C6—C5	0.97 (17)	C11—S2—C10—N2	−178.59 (13)
C2—C3—C6—C7	0.9 (2)	N3—N4—C11—S2	0.73 (16)
S1—C3—C6—C7	−177.84 (12)	N3—N4—C11—S3	−176.86 (10)
C4—C5—C6—C3	−0.9 (2)	C10—S2—C11—N4	−1.29 (12)
C4—C5—C6—C7	177.88 (15)	C10—S2—C11—S3	176.53 (10)
C8—N1—C7—C6	−174.53 (12)	C12—S3—C11—N4	−16.16 (16)
C1—N1—C7—C6	−50.20 (16)	C12—S3—C11—S2	166.30 (9)
C3—C6—C7—N1	15.4 (2)	C11—S3—C12—C13	−103.33 (14)
C5—C6—C7—N1	−163.23 (14)		

### Hydrogen-bond geometry (Å, °)

**Table:**

*D*—H···*A*	*D*—H	H···*A*	*D*···*A*	*D*—H···*A*
C12—H12B···N1^i^	0.99	2.60	3.473 (3)	147
N2—H2···N3^ii^	0.89 (1)	2.02 (1)	2.902 (2)	171.(2)
C5—H5···O1^iii^	0.95	2.46	3.279 (2)	145

Symmetry codes: (i) −*x*+1, −*y*+1, −*z*; (ii) −*x*+2, −*y*+1, −*z*; (iii) −*x*+3/2, *y*−1/2, −*z*+1/2.
